# Perspective of Liberian surgical care providers on ethical issues in global surgical collaborations

**DOI:** 10.1371/journal.pone.0347519

**Published:** 2026-04-21

**Authors:** Madeleine Kruth, Lemfuka Dieudonné, Farhad Udwadia, Catherine Binda, Emilie Joos, Shahrzad Joharifard

**Affiliations:** 1 Global Surgery Lab, Department of Surgery, University of British Columbia, Vancouver, British Columbia, Canada; 2 Faculty of Medicine and Dentistry, University of Alberta, Edmonton, Alberta, Canada; 3 ELWA Hospital, Monrovia, Liberia; 4 Division of Vascular Surgery, University of British Columbia, Vancouver, British Columbia, Canada; 5 Division of General Surgery, Department of Surgery, Vancouver General Hospital, University of British Columbia, Vancouver, British Columbia, Canada; 6 Division of Pediatric Surgery, Department of Surgery, British Columbia Children’s Hospital, University of British Columbia, Vancouver, British Columbia, Canada; Jhpiego, UNITED STATES OF AMERICA

## Abstract

As the field of global surgery develops, increased attention is being paid to its ethical considerations. However, there is a dearth of research on ethical challenges in global surgery that includes the voices of providers from Low- and Middle-Income Countries. This project seeks to understand the ethical concerns, considerations, and priorities in global surgery collaborations from the perspective of surgical care providers in Liberia. Using purposive/convenience sampling methods, we recruited eight surgical care providers (surgeons [n = 3], nurses [n = 3], and general practitioners [n = 2]). Participants included six males and two females from four different sites in Liberia. All had experience in international partnerships for humanitarian surgical work in both urban and rural locations. Semi-structured interviews were conducted using an interview guide and transcribed using a voice recognition program. Following transcript cleaning and verification, interviews were analyzed using thematic coding with qualitative coding software. Prominent themes included justice, consent, colonial legacies, fairness, sustainability, and system strengthening. Ultimately, this study underscores the ethical complexities of surgical care delivery in low-resource settings and the need for collaborative, context-sensitive, and sustainable solutions.

## Introduction

As the field of global health continues to develop, including growing consensus on the field’s key definitions and priorities [1–5], increased attention is being paid to its ethical considerations [6–9]. These discussions generally assume the foundational principles of autonomy, beneficence, nonmaleficence, and justice, and revolve around challenges in their application to global health provision [10]. While some advocate for a global approach to tackle transnational health challenges such as the climate crisis and pandemics [1,11], others critique the term “global” for its connotations of imperialistic healthcare practices [6]. Short-term medical missions, which have historically dominated the landscape of global health, often come under scrutiny for their negative impacts on host communities [7,12]. Issues like mismatches between perceived needs and local realities, inadequate pre-departure training, and the burdens imposed on host communities by visiting volunteers are well-documented [2,7,12].

Many ethical dilemmas identified in global surgery mirror those identified in other areas of global health. Language and cultural barriers, visitors lacking expertise but perceived as experts, and the erosion of local infrastructure by visiting care providers all complicate the informed consent process and degrade the quality of care [9,13]. Trainees from high-resource settings practicing in low-resource settings often face moral distress due to reduced supervision, practicing outside their regular scope, and navigating cultural differences in medical practice [9,12–14]. These challenges are further complicated by attempts to mitigate the lack of access to surgical care in low and middle income countries (LMICs). Educational exchanges between high-resource and low-resource settings may exacerbate “brain drain” in local communities and perpetuate resource shortages [9,15]. These challenges underscore tensions involving the fundamental principles of medical ethics, including patient autonomy, justice, beneficence, and nonmaleficence [16]. In recent years, there has been a shift towards more sustainable and collaborative approaches in global health interventions [17]. These approaches have emphasized bidirectional educational exchanges and systems-level interventions [7,8,11]. However, in fields like global surgery, short-term missions and surgical camps remain prevalent [11,14,15].

While several ethical challenges have been identified, it is crucial to consider *who* is driving this discourse. A scoping review in 2020 revealed that most of the literature discussing ethical considerations in global surgery originated from authors in High Income Countries (HICs) [9]. This research highlights the pressing need to amplify the voices of surgical care providers in LMICs. Acknowledging the vastness, diversity, and value of these voices, this study aims to begin to respond to the dearth of LMIC perspectives in this discourse by intentionally centering the perspectives and priorities of surgical care providers in Liberia.

Liberia, a West African nation with a population of 4.8 million, has faced immense challenges in its healthcare system due to a prolonged civil war and devastating Ebola epidemic [18,19]. The country is ranked 177 out of 191 on the UN Human Development Index, and depends heavily on foreign support to respond to domestic challenges such as infrastructure development and healthcare delivery [20,21]. As a result, Liberia has become a focal point for partnerships with international humanitarian organizations from abroad [20]. Our study seeks to explore the understandings of ethical issues and priorities of Liberian surgical care providers who have experience working within global surgical partnerships. Through this exploration, we aim to contribute to a more nuanced understanding of ethical practices in global health, grounded in the realities and perspectives of those directly involved in providing surgical care in LMICs.

## Methods

### Study design

The qualitative design of this study was reflexive; while it broadly included logical progression of stages from problem formulation to generation of conclusions, (as described by Maxwell in *The SAGE handbook of applied social research methods*, p. 214), it also allowed for development and modification of both theory and data interpretation synchronously. Due to the experiential nature of the question we were attempting to answer (“What are Liberian surgical care providers’ perspectives on ethical issues surrounding global surgery initiatives?”), we elected for a qualitative design with a phenomenological approach, which allows for the investigation of experiences’ from individuals’ perspectives [22,23].

### Ethics approval

Prior to recruitment activities, ethics approval for this study was granted by the University of British Columbia’s Office of Research Services (application H21-01372).

### Participant recruitment

#### Sampling strategy and inclusion criteria.

Using purposive and convenience sampling methods (e.g., purposeful selection of interview candidates based on the probability of generating information-rich interviews for further analysis, and engagement with participants based on their availability for interview) [24], surgical care providers working in Liberia who had been involved with global surgery partnerships were invited to participate.

#### Recruitment procedures.

Recruitment was completed through contacting surgical care providers known to the second (LD) and senior authors (SJ) over email, and was conducted from February 1-August 1, 2023. Following informal introduction to the project by the second author (LD) to gauge interest in participating, the first author (MK) then shared formal initial contact and consent materials with amenable participants.

### Data collection

#### Recruitment.

Over the interview period, twelve surgeons (general, orthopedic, pediatric), seven nurses (scrub nurses, operating room nurses, nurse anesthetists), two general practitioners, two administrators, and one sterile processing expert were contacted and invited to interview. Efforts were made to ensure diverse gender, professional, and geographic representation of recruited participants. Of the number contacted, 41% expressed interest in participating and 29% (8 respondents) completed interviews. Appropriate sample sizes for qualitative research in the literature varies, but is typically between 6–12 interviews [25]. Although the initial goal of 12 interviews was not met, thematic saturation was achieved. This was determined by the paucity of new themes emerging after the seventh interview was analyzed using a bootstrapping technique (7 + 1 yielding ≤5% novel themes) [26], further described below. Given the achievement of thematic saturation further interviews were not sought out.

#### Interview procedures.

Prior to initiating interviews, informed written consent was obtained. Participants were provided both physical and electronic copies of a written consent form and were given opportunity to ask questions to both the contacting researcher (LD) or of the primary interviewer (MK) via email. Participants signed the consent on hard copies, which were then scanned and stored on a password-protected, encrypted drive. At the onset of interviews, the interviewer further confirmed the participants’ understanding of the project, willingness to participate, and outlined the withdrawal process following the interview. Undue influence on participation relating to professional relationships between the second author (LD) and participants was avoided by providing participants opportunities to withdraw prior to commencing the interview during verbal confirmation with the interviewers (MK, CB), as well as the option to withdraw following completion of the interview. Completion status of participant interviews was not shared with any of the team members other than the primary interviewer (MK), and participant responses were anonymized prior to team coding, allowing potential conflicts of interest to be avoided.

Participants were interviewed by an interviewer (MK, CB) over a secure voice or video messaging platform (Zoom, WhatsApp). Interviews followed a semi-structured approach based on an interview guide (Table 1, described below). On average, interviews took approximately forty-five minutes to complete.

**Table 1:** Interview guide.

Section 1 – demographics and practice
*What is your age?* *What is your professional role?* *What does a typical day of work look like for you?* *What are some of the challenges to providing care in your community?*
**Section 2 – experience with global surgical initiatives**
*Tell me about the global surgical initiatives or partnerships that have been set up in your community*
Follow up questions:
• *How involved were you in these programs?*
• *Who else was involved?*
• *What were the main goals of the initiative?*
• *Was the local community consulted in terms of goal or objective setting?*
• *Was/were the hospital/healthcare providers consulted?*
• *How do you think the goals of the project responded to the needs of the local practitioners and hospital? The needs of the community?*
**Section 3 – outcomes and ethical challenges**
*Do you have any initial thoughts you’d like to share in regard to the ethical challenges of the initiative you’ve told me about?* Follow up questions:
• *What were the outcomes of this initiative?*
• *What were some of the challenges or shortcomings?*
• *Did this initiative leave your community with better surgical care than before?*
*• What were some of the ethical challenges you encountered?*
Follow up questions:
• *Did the initiatives have a lasting impact? Were they sustainable in the long run?*
• *How were the interpersonal interactions amongst professionals? Were there any challenges associated with language, culture, expectations, etc.?*
• *Did you feel appropriately compensated/paid for the work you have done?*
• *Did you feel you were recognized for your work?*
• *Did the outcomes respond to the needs of the community? Did the outcomes respond to a need you have as healthcare professionals?*
**Section 4 – concluding questions**
*Is there anything else you would like to share about your experience?*
*If you were to take part in designing a future global surgical initiative/partnership, how would you approach it?*
Follow up questions:
• *Are there things you would do differently?*
• *Are there things you would do the same?*
*• What would you like to see done with the results of this study?*

#### Interview guide development.

The interview guide was semi-structured in design, and was divided into four sections: (1) provider demographics and scope of practice; (2) experiences with global surgical partnerships; (3) barriers and facilitators of past partnerships and ethical challenges identified; and (4) concluding questions (see Table 1). Once rapport had been developed between the interviewer and the participant, the format allowed for exploration of themes that emerged during the interview. The first two interviews served as pilots, following which the first, second, and senior authors modified the interview guide as per feedback obtained during these interviews. We maintained this process of peer debriefing throughout the coding process in order to ensure credibility.

### Data analysis

#### Transcription.

Once completed, interview transcripts were uploaded onto a transcription software (Otter.ai) for initial processing. Audio files of the interviews were uploaded to the secure online transcription provider, rather than transcribed synchronously. Rough transcripts were produced, which were then verified and cleaned manually, and identifying details removed. Final transcripts were then uploaded onto a qualitative coding software (NVivo QSR 12) for thematic analysis. The use of Otter.ai for this method of transcription has been approved by the research ethics boards of the main institutions of the researchers, and is compliant with health information privacy laws in Canada, where research approval and transcription took place.

#### Coding process.

A codebook (Table 2) was drafted based on a preliminary literature review and the lived experience of the second author (LD). This codebook was tested with the two pilot interviews. Analysis was conducted in iterative fashion following establishment of the codebook, which provided a framework for overarching coding goals. Over two passes, the transcripts were analyzed for both themes identified in the preliminary codebook, as well as for emergent themes which were either nested under existing codes or resulted in the creation of new codes. This iterative process was documented and checked by other team members over each pass, in order to ensure that the coding process was dependable.

**Table 2:** Codebook.

Nodes	Codes	Sub-codes
Demographics	Age Gender Location	
Profession	Surgeon	Specialty surgery
	General practitioner/ other practitioner	
	OR staff	Nursing (scrub nurse, nurse anesthetist)
		Anesthesia
		OR technician
	Administrators	
Liberian work experience	Sites Time spent in current profession Daily activities/duties Experience with international surgical initiatives	
Outside work experience	Work or training outside Liberia	
Challenges to providing care	Limitations on	Equipment
		Infrastructure/setting
		Consumable supplies
		Staff on duty per day/per OR
	Lack of specialized care	Specific specialties
		Lack of opportunities for further training
	Patient population	High volume (Demand-supply mismatch)
		Level of education of population receiving care
		Adherence to the care
Challenges to surgical partnerships	Long-term sustainability	Lack of capacity building
		Lack of follow-up
	Logistics	Coordinating between local and international teams
		Patient communication about timing of outreach
		Number of patients exceeding capacity of program
	Ethical challenges	Difficulty with resource allocation
		Lack of overall supplies
		Conflicts of interest
		Informed consent (formal and informal)
		Compensation for work
		Recognition for work
	Cultural challenges	Work habit
		Inter-personal
		Language
		• Challenges communicating expectations to patients
		• Challenges communication between local and international professionals
	Expectations (match or mismatch)	Patients receiving care
		Local professionals
		International professionals
		Administration
Lessons learned	Positive impacts	Relationships within community
		• Consulting/input/direction from local community
		• Employing/utilizing local community
		• Training local community
		Sustainability focused
		Importance of local buy-in/direction
		Relief on burden of disease (patient outcomes)
	Suggestions for improvement	Relationships within community
		• Consulting/input/direction from local
		• community
		• Employing/utilizing local community
		• Training local community
		Sustainability focused
		Importance of local buy-in/direction
		Relief on burden of disease (patient outcomes)

#### Thematic analysis.

Interviews were analyzed using conventional thematic qualitative analysis, a descriptive method of reducing quantitative datasets through the assignation of codes to transcripts to identify and analyze patterns of meaning [27,28]. This method of analysis was chosen as the goal of this project was to elucidate the nature of Liberian surgical care providers’ conceptualization of the ethics of global surgical partnerships, to which thematic analysis is well-suited [28].

### Trustworthiness and rigor

To establish trustworthiness, the criteria established by Lincoln and Guba (1985) of credibility, transferability, confirmability and dependability were used, as recently described in their application to qualitative health systems research by Ylitörmänen, Kvist & Turunen [29].

#### Credibility, transferability, dependability and confirmability.

Interviews were conducted primarily in English by the first author. While this is the lingua franca of Liberia, it was not necessarily the first language of the participants; this reality may have results in misinterpretations, reducing the study’s validity. However, due to the open-ended nature of the interview guide, the interviewers were free to ask for clarification throughout, and often used techniques of repeating what was understood in the moment to verify appropriate interpretation. The interviewers also built trust with the participants by engaging in discussion with the respondents both prior to the initiation of the interviews, informing participants about themselves and the interview process and giving them opportunity to ask questions or seek clarification; and following the interview, to ensure the participants had the opportunity to communicate anything not covered in the interview, to strike comments, or to fully withdraw. Credibility was also ensured throughout the interview process through provision of as much time was needed for participants to respond to questions, and by following up on points raised. It was also ensured by offering participants the option to stay informed on the progress of the study, including receiving copies of any emerging publications. Confirmability was ensured through the utilization of established analyses process and in transparency among the research team, as well as in the use of examples to illustrate the designation of meaning to the codes. Lastly, discussion of the data and the categorization process among the research team members contributed to dependability, and utilization of established methods for qualitative data analysis as well as clear and accurate descriptions of the data collection and analysis allowed for transferability.

### Reflexivity and researcher positionality

#### Impact of research team backgrounds and training.

There is a possibility that the decision to participate in interviews was influenced by the position of the recruiting authors (a senior surgeon in Liberia and a Canadian surgeon who has been heavily involved in global surgical outreach programs in Liberia), especially in the case of more junior staff. All efforts were made to mitigate this possibility, through the informed consent process, the interviews themselves, and in the opportunity for withdrawal after interview completion. Specifically, the interviewers were both junior trainees (medical students) at the time of interview, which may have alleviated the possibility of some of the hierarchical pressures that can be present with research in the field. Participants were also given the opportunity to withdraw prior to, during, or following interviews, and the second and senior authors were not informed as to interview completion status of participants. This allowed for the anonymization of interview transcripts and protection of participants from any concern regarding professional repercussions.

#### Potential biases and assumptions.

Interviews were conducted in English; while this is the official language of Liberia, it was not the first language of the majority of the participants. It is possible that the intended meaning of the participants’ responses was not fully appreciated by the interviewers.

## Findings

### Participant demographics

Participant demographics are described in Table 3. The majority of participants recruited were male (n = 6). While we did not have equity in the genders of our participants, the demographics were reflective of the general pattern of gender distribution of surgeons and nurses in the region [30,31]. We did obtain diversity in age and professional background, as described in Table 3.

**Table 3:** Participant demographics.

Demographic	Responses
Age (years)	28–60
Gender	Man (n = 6)
	Woman (n = 2)
Country of origin	Liberia (5)
	Other Sub-Saharan African country (2)
	HIC (1)
Profession*	General practitioner (n = 2)
	Surgeon (n = 3)
	• Pediatric surgeon (n = 1)
	• General surgeon (n = 2)
	Nurse (n = 3)
	• Surgical nurse (n = 2)
	• Nurse anesthetist (n = 1)
	Supervisor/ director (n = 2)

* Two participants held more than one professional role.

### Justice

Participants described justice as a guiding but often unrealized ideal in the provision of surgical care in Liberia, particularly within the context of global surgical collaborations. Across interviews, they emphasized justice as a structural concept—one concerned with equitable access to surgical services, fair distribution of resources within the health system, and the ability of providers to meet patients’ needs in conditions of profound scarcity. However, participants consistently noted that these goals were constrained by the structural realities of their health system. The context of surgical care was characterized by extreme demand, resource scarcity, and a strong moral commitment among practitioners who felt both the weight of duty and the inequities of their environment.

Participants described overwhelming workloads resulting from high patient volumes and limited staff. Participant 5 noted, “Our day is hectic […]. Because we have to provide surgical care for populations higher than we can actually handle […] The surgeon has the chance to live on a compound of the hospital. And he’s called upon 24/7.” The sense of moral obligation was strong despite these challenges. Participant 2 explained: “I always love to come around, because I know that we are insufficient in the country […] I’d be energized when I’m going to work, I feel so encouraged because […] we choose to do so. And with our country coming from war, with a limited number of […] human resource, we need to give our best.”

Justice was frequently discussed through the lens of material deprivation and the broader structural conditions in which both local providers and international collaborators operate. The lack of resources—consumables, anaesthetic agents, equipment, and reliable infrastructure—was identified as the most persistent threat to equitable surgical care. These shortages translated directly into inequities in access. Because patients were responsible for purchasing their own surgical supplies, many were excluded from care due to cost. Participant 2 emphasized that “The issue of getting the drugs sometimes is still really a challenge for us. For those drugs are […] very expensive.” In rural areas, this injustice was compounded by poverty and limited market access: “You tell [the patients] you have to go and buy this… it is sometimes frustrating for them. It delays initiation of care for the patients.” (Participant 7)

Understaffing was a near-universal concern, raising both moral and practical challenges for providers attempting to maintain equitable standards of care. Participant 6 described situations in which “only two anaesthetists” were available to cover three operating rooms, leaving interns to monitor patients. Participant 2 elaborated that this scarcity sometimes created ethical dilemmas: “I will be sometime obligated to give care to my own relatives, which will create some level of anxiety for me. It’s not supposed to be so, but because we lack […] the professional people that are supposed to do it.” Infrastructure limitations further constrained justice in surgical care. Frequent power cuts and water shortages disrupted operations and compromised safety. “We do not have constant supply of electricity,” said Participant 3. “And so sometimes we have a break in the electricity while surgery is ongoing.” Similarly, Participant 6 recalled, “There will be days where we will have issues with water in the operating room.”

Within this context, justice was understood as a systemic aspiration – one that global surgical partnerships often seek to address but that remains difficulty to achieve in practice. Despite these constraints, participants consistently emphasized their ethical resolve. As Participant 5 summarized, “We try to navigate all of [those challenges] every day to make sure that our patients get the care they deserve.” Justice, therefore, was not only an institutional aspiration but also an individual moral practice enacted daily in conditions of scarcity.

### Consent

Participants described consent as a complex ethical process shaped by communication, cultural context, and patients’ understandings of surgical care. Unlike justice or fairness, which participants discussed primarily in relation to structural resources or the distribution of opportunities, consent was experienced as an interpersonal and communicative challenge in clinical encounters – one that became particularly complicated in the setting of global surgical collaborations. Patients’ hesitancy toward surgery was often grounded in fear, mistrust, and limited comprehension of medical procedures. Participant 2 explained that “Many patients and their relatives didn’t really understand the consent. […] So […] most of them were afraid [of surgery]. And they were saying, ‘Oh, we cannot come. We don’t want to be a part [of it].’” For providers, the challenge was not merely linguistic but epistemic: the biomedical concept of consent was not always easily translated into patients’ local contexts or ways of understanding illness and treatment.

Participants linked these communication difficulties to broader sociopolitical and economic conditions that shape surgical decision-making in global surgical partnerships in Liberia. Participant 1 reflected that “The political context and the economic context in Liberia are chronically challenging […].” Although surgical procedures themselves were often funded under the public system, chronic underfunding meant that the associated consumable materials were not. As a result, the act of consenting to surgery often implied accepting a substantial financial burden. In a largely out-of-pocket system, this economic reality complicated the meaning of consent for many patients. As Participant 8 explained, “People don’t afford the cost of the surgery because […] there is no coverage.”

Within global surgical partnerships, these challenges took on additional dimensions. Patients often encountered foreign providers for the first time during visiting surgical outreach programs, sometimes with limited understanding of who these teams were or how care would be followed up after interventio. Participants expressed concern that consent under such circumstances risked being procedural rather than informed. For them, meaningful consent required trust built over time, which required stable, transparent, and ongoing relationships between providers, patients, and communities.

### Colonial legacies

Participants’ reflections revealed the enduring imprint of colonial dynamics within global surgical partnerships. Although many valued the educational and material contributions of visiting teams, they also described asymmetries in power, decision-making, and recognition that mirrored colonial geopolitical hierarchies.

Goal-setting and planning processes were often externally driven. As Participant 5 observed, “When these surgical outreaches are set up, the local communities are *informed*. They are *informed* about that outreach.” In practice, however, “being informed” did not equate to having influence over the direction or priorities of the project, and instead implied *post-hoc* engagement in project implementation. Several participants also expressed frustration at the opacity of partner planning. Participant 3 noted, “You cannot impose on the partners. You don’t know their work plan. They know their own plan.” This lack of agency was experienced as a subtle form of disempowerment—replicating extractive relationships where local expertise was undervalued or tokenized.

The pattern extended into knowledge exchange. Participant 5 described how “we normally have a lot of surgeons visiting… sometimes they learn from us, sometimes we also learn from them.” Yet they went on to note that reciprocity was uneven: “most of the cases we see are hernia cases. So, we taught them how we did hernias here. And they benefit from us mainly.”

Economic inequities further reinforced these hierarchies. All participants reported that they were not financially compensated for their participation in surgical outreach programs, while visiting partners received stipends or institutional support. Participant 6 recounted: “Most of the time financial compensation is not taken in consideration… international members of the team were compensated financially, but the local team… people will just ignore and say […] we are helping patients.” This disparity occasionally fostered resentment among local staff who carried heavier workloads during these partnerships. “My nursing team,” the participant continued, “were like okay, we are working more and more compared to what we had as workload before, but why is it that we cannot get financial cooperation or encouragement?”

Nevertheless, participants described moments of shared humanity and mutual appreciation that challenged this imbalance. Community gratitude—manifested through “a lot of community members coming to hug you and say thank you” (Participant 5)—offered symbolic recognition even when material compensation was absent. Still, the underlying inequities pointed to a lingering colonial structure in which authority, resources, and decision-making largely resided with visiting actors.

### Fairness

While participants discussed justice primarily in terms of structural equity within the health system, fairness emerged as a related but distinct ethical principle concerned with the distribution of opportunities, resources, and responsibilities within everyday clinical practice and in relationships with global surgical partners. For participants, fairness referred less to the overall structure of the health system, and more to whether interactions – between providers, patients, institutions, and international collaborators – felt balanced and respectful in practice. In both routine surgical care and global surgical partnerships in Liberia, participants described fairness as essential and yet often difficult to sustain..

At the local level, fairness was challenged by the uneven geography of surgical access. Rural facilities operated with fewer staff, weaker infrastructure, and limited supplies, which constrained their ability to provide timely and safe surgery. Participant 7 described the moral distress of working under such conditions “You have to make a list for the patient to go and get before you can work. In cases of emergency, they can really be strenuous for us.” Requiring patients to purchase their own consumables—such as sutures, gloves, or anaesthetic drugs—placed the burden of resource scarcity on those least able to bear it. In this sense, fairness was experienced as the daily negotiation of how limited resources were allocated among patients who all required care.

Global surgical collaborations also shaped participants’ perceptions of fairness. Outreach programs and visiting surgical teams sometimes temporarily alleviated resource shortages by providing equipment, supplies, or free surgical services. Participants acknowledged the positive impact of these interventions. Participant 7 spoke of children who underwent cleft repairs and were “being pointed to” before surgery, but afterward “their parents are relieved of that burden.” For these families, free access to surgical care represented a powerful form of fairness realized—alleviating suffering and restoring dignity.

However, participants also emphasized that such fairness was often temporary and dependent on the presence of external programs. When supplies were exhausted or outreach programs concluded, the underlying inequities quickly reemerged. As Participant 5 observed, “Sometimes the supplies they bring are short and the hospital has to make available money to buy more supplies. And when that happens […], we do not have budgets for operating at all, and it becomes a problem for the hospital.” This cycle left local providers balancing gratitude for short-term relief with frustration at the absence of structural change.

For participants, fairness was therefore not a static condition but an ongoing relational process —one shaped by scarcity, partnership asymmetries, and their commitment to ensuring that patients benefit meaningfully from both local care and international collaboration.

### Sustainability

Sustainability was described by nearly all participants as the most pressing ethical concern in global surgical engagement. While visiting surgical teams were praised for providing valuable short-term relief, participants emphasized that these efforts rarely translated into lasting improvements in the health system. They viewed sustainability not only as continuity of care, but as the capacity to maintain and build upon surgical services long after visiting teams had departed. Participant 6 summarized this tension succinctly: “I think that those [partnerships] are punctual interventions. And when you have these kinds of interventions […], which are not sustainable in the long term, they don’t solve the issue.”

This lack of continuity left participants feeling caught in cycles of progress and regression. Outreach programs temporarily alleviated surgical backlogs, but the burden quickly returned once they ended. As Participant 5 explained, “This program helped the community […] but we have a lot of surgical patients. So after one, two months, you see—patients full again.” Sustainability, therefore, required more than episodic campaigns; it demanded investment in systems and capacity capable of meeting ongoing needs.

Participants often contrasted the abundance of resources during outreach periods with the scarcity that followed. Visiting teams frequently brought supplies, instruments, and consumables, which provided short-lived relief from daily shortages. “Sometimes not even all of those tools are used on the patients directly,” said Participant 7. “It sometimes helps relieve the burden of not having gloves available, not having swabs available, not having sanitizers available.” However, these temporary contributions did not address the underlying structural deficits that perpetuated dependence. In some cases, donated equipment sat idle due to lack of supporting resources. Participant 2 described receiving anaesthesia machines that could not be fully utilized: “They gave us anaesthesia machines. However, you also need anaesthetic drugs.” Without parallel investment in supply chains and infrastructure, such donations did not have transformative capacity.

Participants also raised concerns about follow-up and accountability. Many visiting teams did not remain long enough to manage postoperative complications or to ensure that patients had access to continued care. This discontinuity, they noted, could undermine community trust and professional confidence. Sustained collaboration—rather than isolated missions—was seen as ethically necessary for both patient safety and system learning.

Some participants envisioned a model of sustainability rooted in repetition and local partnership. Participant 4 emphasized the value of ongoing relationships, explaining, “We told them that […] we need for them to come every year.” For others, sustainability meant capacity building—training local staff and strengthening systems so that influx of foreign professionals to help respond to surgical need became progressively less necessary. Participants also recognized that achieving sustainability involved long-term planning, government engagement, and policies that could integrate outreach programs into national health priorities. Ultimately, sustainability was articulated as both a practical and moral imperative. Participants deeply valued the expertise and resources of visiting teams but urged a shift toward approaches that empowered local providers, ensured continuity of care, and embedded equity into the fabric of the health system. In their view, sustainable global surgical partnerships meant building the capacity for self-sufficiency.

### System strengthening

Participants repeatedly returned to the idea that the ultimate ethical aim of global surgical engagement should be to strengthen Liberia’s surgical system as a whole. They emphasized capacity-building, long-term collaboration, and the development of clear regulatory frameworks as cornerstones of ethical practice.

Participant 2 proposed a phased, collaborative model:

“When [the team] comes, they can work with the people on the ground to search in what part of Liberia that this surgical outreach will be necessary. And we all can figure out and select the appropriate place, that everyone, patients, will have the benefit. Then I think with that we can move on the second phase, how to do it in, you know, empowering the people that use those [surgical skills] in Liberia, empowering them with knowledge and skills.”

System strengthening, for participants, required moving from project-based interventions to institutional partnerships grounded in mutual accountability. Participant 1 reflected on the challenge of defining community needs: “We thought that we knew what the population wanted. It may still be challenging on who will define the need. Do we go at the base and ask them as the population from house to house? Or do we rely on the local leaders or the health professionals to define it?” Such questions revealed a growing recognition that sustainable system strengthening must be participatory, inclusive, and context-specific.

Participants also called for clearer ethical and operational guidelines. They envisioned frameworks that would ensure longitudinal access to supplies, structured training for local providers, and adaptive outreach models responsive to changing population needs. As Participant 1 summarized, “One of the components of health care system strengthening around the world is building robust programs that, in the long term, address the changing needs of a population.”

This vision extended beyond the immediate ethics of surgical campaigns to a broader aspiration for sovereignty in health system development. For participants, genuine partnership meant shifting from dependence on external missions to empowerment of local providers and institutions. In this sense, system strengthening was both a practical and moral project—one that sought to realize justice, fairness, sustainability, and system strengthening not as separate ideals, but as interdependent pillars of ethical global surgery.

## Discussion

This study captured the experiences and ethical reflections of eight Liberian surgical providers across multiple disciplines, offering insight into how global surgery partnerships are perceived by the LMIC partners. Participants described their professional commitment to patient care and their pride in delivering high-quality surgery despite chronic resource limitations. Yet, their narratives also illuminated tensions—between partnership and dependence, generosity and inequity, and short-term gain and long-term sustainability. When examined through the lens of justice, consent, colonial legacies, fairness, sustainability, and system strengthening, participants’ reflections reveal not only the ethical complexity of global surgery partnerships but also the enduring resilience and agency of local surgical providers.

### Justice

Justice emerged as a central ethical concern underpinning nearly every aspect of participants’ reflections. While global surgery partnerships were often motivated by altruism and a desire to address surgical inequities, participants highlighted how the form of these interventions could reproduce inequity. Episodic surgical “camps” brought short-term relief but often left structural disparities untouched, and sometimes worsened them by diverting patients and staff from routine local services.

These findings echo existing critiques that justice in global surgery cannot be reduced to the volume of operations performed, but must instead encompass distributive fairness and procedural equity [7–10]. Participants’ accounts illustrate how justice requires careful balance: meeting immediate surgical needs while simultaneously strengthening the capacity of the local system to serve all patients over time. Injustice was most visible when foreign-led teams operated in isolation, determined priorities unilaterally, or created dependency through patterns of resource donation without integration.

Justice in global surgery thus extends beyond the equitable allocation of surgical care to include fairness in decision-making, access to opportunities for training, and the equitable sharing of risks and benefits between partners. As participants noted, the most ethical partnerships were those that actively listened to local priorities, built mutual accountability, and centered vulnerable populations rather than institutional prestige or efficiency metrics.

### Consent and autonomy

Participants underscored that ethical surgical practice requires not only technical competence but also deep cultural sensitivity and respect for patient autonomy. Informed consent—an ethical cornerstone of medical care—was frequently challenged by linguistic barriers, limited patient literacy, and differing cultural understandings of illness and authority. As several participants described, obtaining meaningful consent demanded time, trust, and contextual understanding, which were often constrained during short-term outreach visits. These challenges parallel findings from broader global health literature showing that consent processes in cross-cultural contexts are frequently shaped by power asymmetries and structural dependency [9]. Visiting teams may unintentionally assume compliance where true understanding is absent. Ethical partnership thus requires deliberate adaptation of consent practices to local norms, as well as collaboration with community leaders and translators to facilitate mutual understanding.

Autonomy was also discussed at the institutional level. Local surgeons often felt that international partners set goals and objectives without full consultation, thereby limiting local decision-making. Rather than shared planning, local communities were “informed” of the partnership’s terms, reflecting a paternalistic pattern reminiscent of colonial health interventions. Reimagining autonomy in global surgery therefore means extending it from the individual to the collective—ensuring that local institutions retain control over priorities, governance, and implementation.

### Colonial legacies

While few participants explicitly invoked colonialism, their accounts resonated deeply with its enduring structures. Participants described asymmetries in authority, decision-making, and recognition, often shaped by resource dependency. Visiting teams frequently held control over surgical priorities and research agendas. These dynamics mirror broader critiques that global surgery can inadvertently reproduce hierarchies of knowledge and power rooted in colonial histories [32–35].

In their recent paper, Qin *et al*. (2024) describe five categories of non-merit based inequalities in global surgery: Western epistemology, geographies of inequity, unequal participation, resource extraction, and asymmetric power and control. Participants highlighted direct experience with all of these categories, from the centering of surgical outreach programs in the Global North in terms of resources and knowledge; to the disproportionate control held by HIC partners over priority setting, knowledge production, funding, and standards creation, The persistence of such patterns highlights the need for intentional decolonization of global surgery. Decolonization in this context is not merely symbolic but structural—it involves dismantling dependency relationships, reforming authorship and funding practices, and redistributing authority to ensure that local voices drive the direction and evaluation of partnerships.

Community-based participatory research and co-leadership models have been proposed as vehicles for decolonizing partnerships [33,35]. Such models align with participants’ calls for engagement that values lived experience and local expertise as forms of knowledge. As one participant emphasized, sustainable partnerships require “planning together,” not “being told.” Meaningful inclusion, therefore, is not an ethical add-on—it is the ethical core of equitable collaboration.

### Sustainability and system strengthening

Participants repeatedly emphasized that sustainability is the ultimate ethical benchmark of global surgery. While sustainability and system strengthening are not in themselves traditional categories in bioethics, more recently there has been a shift towards the ethical imperative of sustainability in health systems [36,37]. The experiences of our participants mirror this – temporary missions, while beneficial in the short term, were viewed as insufficient and even counterproductive when not embedded in broader system strengthening. The abrupt end of outreach programs often left communities with renewed surgical backlogs, idle equipment, and disrupted care continuity.

In their recent paper examining surgical transitions in LMICs, Bakker *et al*. (2025) describe how evolution of surgical systems reflect socioeconomic development of a country. When understood as a challenge relating to surgical volume, Bakker *et al.* argue that system strengthening must be viewed in the broader context of societal and economic development [38]. For our participants, system strengthening involves aligning partnerships with national surgical plans, supporting training pipelines, and integrating external resources into existing governance structures. Participants viewed this not as an optional ideal but as the measure of whether a partnership truly “does good.”

Building capacity within Liberia’s surgical system—through education, research infrastructure, and leadership opportunities—was seen as the only way to ensure that global surgery partnerships leave a lasting ethical footprint. Participants described system strengthening as both a moral obligation and a pragmatic necessity. Short-term missions could alleviate suffering but could not transform the structural deficits driving surgical inequity—shortages of specialists, fragile supply chains, and weak referral systems. The ethical development of global surgery, therefore, lies in shifting from episodic service delivery to long-term institutional partnerships.

### The missing theme: Research

Participants were focused on clinical care, patient experiences, and outcomes, and did not specifically mention research in interviews. However, knowledge translation and dissemination are key components of global health partnerships and require particular scrutiny. Grant *et al*. (2020) emphasize that ethical global surgery research must not only meet international standards but also address questions of local relevance, benefit participating communities, and promote local capacity for future research [9]. Further investigation is needed to explore Liberian surgical care providers’ perspectives on these issues, including how best to share outcomes with host communities.

### Guidelines and frameworks

Participants recognized that global surgery partnerships are inherent to improving quality of care in their settings and emphasized the need for robust guidelines and frameworks to ensure their ethical success. One such framework is the Framework and Evaluation Metrics for Sustainable Global Surgical Partnerships, created via a Delphi process engaging multidisciplinary providers from all WHO regions [17]. Of the six pillars of sustainability identified in this framework, (1) stakeholder engagement, (2) multidisciplinary collaboration, (3) context-relevant education and training and (5) multisource funding were explicitly mentioned by participants as being key drivers of successful partnerships. Additionally, certain checklist items directly correlate with asks from our respondents, such as “Has a community-led needs assessment been conducted prior to the start of this project?” [17]. As such, global and widespread implementation of this checklist, on both high and low resource partners side, could potentially improve ethical gaps identified in this study.

Recently, a narrative review of the “Leave no One Behind” principle proposed an International Certification Program for all global health interventions [39]. This ambitious framework includes criteria such as relevant Sustainable Development Goals and alignment with ethical frameworks, such as Leave No One Behind, and proposes monitoring and evaluation metrics to ensure accountability. While the authors suggest an incentives mechanism for countries meeting the certification criteria, the governance of such a program remains nebulous. Although the co-creation of a list of comprehensive recommendations for the ethical implementation of global surgical partnerships was beyond the scope of the current study, it is possible that future work will involve this type of synthesis.

### Limitations

Limitations include the small sample size and absence of data triangulation. While this study consists of a relatively small sample size, the perspectives gained are a valuable contribution to the understandings of the impacts of global surgical partnerships and their ethical challenges, from the perspectives of surgical care providers from LMICs. Furthermore, we recognize that study recruitment was based on the existing professional relationships of the second and senior authors, which could potentially influence the way in which respondents would broach answers related to partnerships involving associated institutions. This limitation was mitigated throughout the interview and analysis process, including establishing trustworthiness, anonymizing participant responses, and through the positionality of the interviewers as more junior trainees. Finally, due to the nature of our data collection and sample size, we could not conduct a fulsome analysis of differences in perspectives of participants of different genders or professions. This exploration represents a future area of research.

## Conclusion

The aim of this study was to address the gap in research that features the voices of surgical care providers from LMICs in discussions surrounding ethical aspects of global surgical partnerships. Recognizing the diversity and breadth of care provision across LMICs, this study did not attempt to offer a comprehensive or representative account of all perspectives, as significant differences undoubtedly exist across geopolitical contexts. Nevertheless, our findings highlight the ongoing need for deeper understanding of ethical challenges in global surgical partnerships—and for meaningful efforts to begin addressing them.

Future research will involve continued exploration of these themes in collaboration with surgical providers from other LMICs, with the aim of gaining further insight into the complex dynamics that shape partnerships between HICs and LMICs. Ultimately, this study underscores the ethical complexities of surgical care delivery in low-resource settings and the need for collaborative, context-sensitive, and sustainable solutions. By acknowledging and addressing these ethical concerns, stakeholders can move toward equitable access to safe and effective surgical care for all, regardless of geography or socioeconomic status.
